# Development of a Genetically Encoded and Potent PDE6D Inhibitor

**DOI:** 10.1002/cbic.202500739

**Published:** 2025-11-18

**Authors:** Atanasio Gómez‐Mulas, Elisabeth Schaffner‐Reckinger, Hanne Peeters, Rohan Chippalkatti, Arnela Dautbasic, Matthew James Smith, Shehab Ismail, Daniel Kwaku Abankwa

**Affiliations:** ^1^ Cancer Cell Biology and Drug Discovery Group Department of Life Sciences and Medicine University of Luxembourg 2 place de l’Université 4365 Esch‐sur‐Alzette Luxembourg; ^2^ The Mechanistic Molecular Biochemistry Group Department of Chemistry KU Leuven Celestijnenlaan 200G 3001 Heverlee Belgium; ^3^ Institute for Research in Immunology and Cancer Pavillon Marcelle‐Coutu Université de Montréal 2950 Chemin de Polytechnique Montréal Québec H3T 1J4 Canada; ^4^ Programmes de biologie moléculaire Université de Montréal Montreal Québec H3C 3J7 Canada; ^5^ Department of Pathology and Cell Biology Faculty of Medicine Université de Montréal Montréal Québec H3T 1J4 Canada

**Keywords:** cancer, inhibitors, PDE6D, RAS, RASopathy

## Abstract

PDE6D is a trafficking chaperone of prenylated proteins, such as small GTPases. Several small molecule inhibitors have been developed against it, given that the oncogene K‐Ras is one of the cargo proteins. Inhibitor development suffers from the fact that inhibitors against the hydrophobic pocket of PDE6D are typically poorly water‐soluble. Herein, the development of genetically encoded inhibitors that are inspired by high‐affinity natural cargo of PDE6D is described. The most potent inhibitor, SNAP‐STI, encodes merely a farnesylated tetra‐peptide, which efficiently blocks PDE6D binding of farnesylated cargo. Direct comparison with small molecule PDE6D inhibitors suggests its higher potency. It is shown that inhibition of K‐Ras membrane anchorage and K‐RasG12C‐dependent MAPK signaling by SNAP‐STI is weak, consistent with what is observed after PDE6D knockdown. The data therefore further support that PDE6D is not a suitable surrogate target for efficient inhibition of K‐Ras membrane anchorage and MAPK‐activity. Nonetheless, by exploiting contacts at the pocket entry, a generalizable strategy to design high‐affinity PDE6D inhibitors is established, providing powerful tools for PDE6D biology and target validation.

## Graphical abstract

PDE6D is a trafficking chaperone of prenylated proteins. Current small molecule PDE6D inhibitors target its hydrophobic pocket, rendering them typically poorly water soluble. Herein, PDE6D binders are generated by combining parts of the K‐Ras4B and INPP5E C‐termini. The genetically encoded high‐affinity PDE6D inhibitor SNAP‐STI is thus generated. Like in natural PDE6D cargo, high affinity is achieved at the entrance of the hydrophobic pocket.

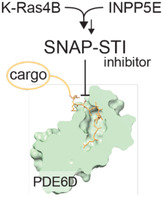

## 1 Introduction

The Ras‐MAPK‐pathway regulates vital cellular processes, including proliferation and differentiation. Its overactivation is associated with cancer and developmental diseases collectively called RASopathies, which affect multiple organs.^[^ ^1^ ^]^ GTP loading switches the conformation of Ras, which initiates downstream signaling by recruiting effector proteins to Ras at the plasma membrane. For example, recruitment of the effector Raf to membrane‐associated Ras initiates the MAPK‐pathway.^[^ ^2^ ^]^ Farnesylation of the CAAX‐box of the hypervariable region provides the affinity of Ras proteins toward membranes. In this motif, the cysteine (C) is prenylated, followed by proteolysis of the AAX‐tripeptide (aliphatic residue A and any residue X) and carboxymethylation. Long‐range diffusion of Ras through the cytosol requires trafficking chaperones, which shield the prenyl moiety. This is necessary to feed the active vesicular transport of Ras to the plasma membrane.^[^ ^3^ ^]^

The trafficking chaperone PDE6D has been proposed as a surrogate target for K‐Ras4B (hereafter K‐Ras).^[^ ^4^ ^]^ It binds the farnesyl‐membrane anchor at the C‐terminus of K‐Ras, but also other prenylated proteins, including dually prenylated small GTPases, including Rab1B, Rab4A, and Rab7A.^[^ ^5^ ^]^ PDE6D is also a major trafficking chaperone of prenylated proteins operating in the primary cilium, such as the lipid phosphatase INPP5E.^[^ ^6^ ^]^ The primary cilium is an antenna‐like membrane protrusion on most stem/progenitor cells where it serves as a hub for several developmental‐signaling pathways, such as Wnt and Hedgehog.^[^ ^7^ ^]^ The distinct localization of PDE6D cargo to the bulk plasma membrane and primary cilium is mediated by the two small GTPases Arl2 and Arl3, respectively. When GTP‐bound, they bind to PDE6D to release low‐affinity cargo, such as K‐Ras (GTP‐Arl2) or high‐affinity cargo, such as INPP5E, in addition to low‐affinity cargo (GTP‐Arl3).^[^ ^6^ ^,^ ^8^ ^,^ ^9^ ^]^

The fact that Ras plasma membrane localization is necessary for its activity led to the development of farnesyl transferase inhibitors (FTI) as the first Ras drug targeting approach. These inhibitors failed in the clinic, due to alternative prenylation of K‐Ras and N‐Ras by geranylgeranyl transferase I, the two Ras isoforms most frequently mutated in cancer.^[^ ^10^ ^,^ ^11^ ^]^ Given that PDE6D facilitates plasma membrane localization of K‐Ras,^[^ ^12^ ^,^ ^13^ ^]^ a number of small molecule inhibitors were developed against PDE6D,^[^ ^10^ ^]^ including our own Deltaflexin1, ‐2, and ‐3.^[^ ^14^ ^,^ ^15^ ^]^ Deltaflexin3 is a highly water‐soluble, low nanomolar inhibitor of the prenyl‐binding pocket of PDE6D. However, during its development, we also noticed that compounds with a higher affinity become less soluble and acquire more off‐target activities.^[^ ^15^ ^]^ Like with other PDE6D inhibitors, its ability to shut down MAPK signaling was surprisingly low considering it indirectly blocks K‐Ras signaling. However, this low activity was consistent with the fact that PDE6D directs only about 25–50% of the plasma membrane trafficking of K‐Ras.^[^ ^15^ ^]^ Moreover, we noticed discrepancies with the reported target affinity values. It is common to establish the in vitro affinity of inhibitors to PDE6D in a competitive fluorescence polarization assay using fluorescently labeled atorvastatin as a PDE6D‐binding probe.^[^ ^4^ ^]^ However, when measuring the affinity with a Rheb‐derived farnesylated peptide or in an SPR assay directly looking at the interaction between K‐Ras and PDE6D, only micromolar affinities were recovered.^[^ ^14^ ^,^ ^15^ ^]^

A way forward in PDE6D inhibitor development could be inspired by its natural cargo, where affinity is modulated not only by contacts within the hydrophobic prenyl‐binding pocket of PDE6D but also by interactions at the pocket entrance. For example, the protein INPP5E binds with K_D_ = 4 nM to PDE6D as compared to K_D_ = 2.5 μM for binding of K‐Ras.^[^ ^6^ ^,^ ^16^ ^]^ This almost 1000‐fold increase is mediated by only two residues at position ‐1 and ‐3 upstream of the prenylated cysteine.^[^ ^5^ ^,^ ^6^ ^]^

## 2 Results and Discussion

**Figure 1:**
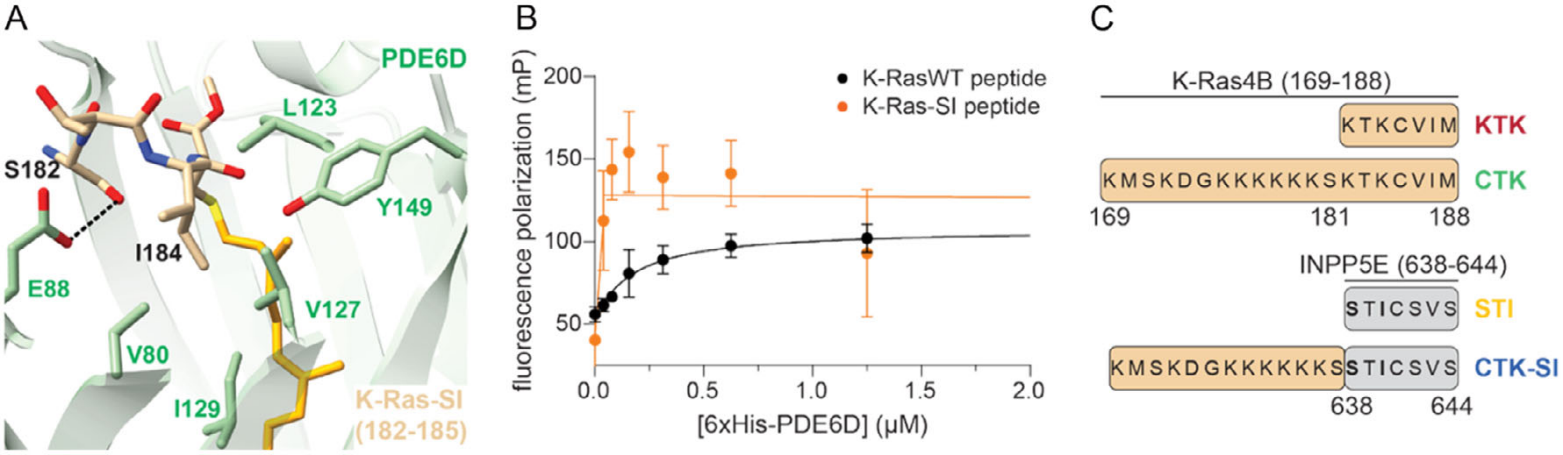
Genetically encoded peptides derived from INPP5E are high‐affinity PDE6D binders. A) Crystal structure of K‐Ras‐SI (only residues 182–185 are shown in tan, farnesyl moiety in orange) and PDE6D (green) (PDB ID: 7Q9U). A hydrogen‐bond between the side chain of Ser182 and Glu88 of PDE6D is indicated by dashed lines. B) Fluorescence polarization binding data of 50 nM wild‐type K‐Ras‐derived peptide (FAM‐KKKKKKSKTKC‐Far‐OMe) and K‐Ras‐SI‐derived peptide (fluorescein‐DGKKKKKKSSTIC‐Far‐OMe) and increasing concentrations of 6xHis‐PDE6D. Means ± SD are plotted of *n* = 3 independent biological repeats. Solid lines represent the quadratic binding equation fit (K‐RasWT peptide) and the two linear equation fits consistent with an active site saturation titration regime (K‐Ras‐SI peptide). C) Names of the genetically encoded PDE6D binders and their sequence composition relative to K‐Ras (tan) and INPP5E (gray).

We aimed at generating genetically encoded, high‐affinity binders to PDE6D by exploiting contacts at the entrance of the prenyl‐binding pocket of PDE6D (**Figure** **1**A). This approach was inspired by the nanomolar binding affinity of INPP5E that is mediated by two residues at the ‐3 and ‐1 position relative to the prenylated cysteine (Figure 1A). Mutating these two lysine residues of K‐Ras to the serine and isoleucine (SI) found at the corresponding positions of INPP5E resulted in the K‐Ras‐SI mutant.^[^ ^5^ ^]^

Fluorescence polarization assays confirmed binding of a farnesylated and carboxymethylated peptide derived from the C‐terminus of wild‐type (WT) K‐Ras to PDE6D with a K_D_ = 0.15 ± 0.05 μM (Figure 1B), consistent with prior reports.^[^ ^17^ ^]^ By contrast, a K‐Ras‐SI‐derived peptide reached saturation rapidly when 39 nM of PDE6D were titrated against 50 nM of the peptide, indicating an affinity far exceeding that of the wild‐type (Figure 1B).

BRET‐biosensors can assay the effective binding or competitive activity of genetically encoded inhibitors in HEK293‐EBNA (hereafter HEK) cells.^[^ ^18^ ^]^ Plate reader‐based BRET experiments can detect binding events inside intact cells following a distance‐dependent (<12 nm) energy transfer between tagged proteins.^[^ ^19^ ^]^ One protein of interest is tagged with a Renilla Luciferase variant (e.g., RLuc8 or NanoLuc, nL), which catalyzes the enzymatic conversion of the chemical substrate coelenterazine 400a to provide the donor energy. Energy is transferred to a close acceptor‐fluorophore (e.g., GFP2 or mNeonGreen, mNG) that is genetically fused to an interaction partner. Using PDE6D‐ and K‐Ras‐derived BRET pairs, we confirmed that the high increase in affinity observed in vitro (Figure 1B) was maintained in the interaction‐dependent BRET of the K‐Ras‐SI mutant (Figure S1A, Supporting Information).

We reasoned that the last seven amino acids of INPP5E (STI‐CSVS, with C representing the prenylated cysteine) represent a minimal sequence for a genetically encoded high‐affinity PDE6D inhibitor. To this end, we genetically fused this sequence via a 15‐residue‐long GS‐linker to the C‐terminus of a SNAP‐tag. In addition, we generated a longer hybrid version where the seven INPP5E residues were N‐terminally extended by residues 169–181 of K‐Ras (CTK‐SI). The matching controls were the corresponding peptides named KTK and CTK, which were entirely derived from K‐Ras (Figure 1C). Note that CTK‐variants describe the 20‐mer polypeptide derived from the K‐Ras C‐terminus, where the designation ‘CTK’ has been established for many years.^[^ ^20^ ^]^

We evaluated these four PDE6D binders in a BRET assay designed to measure the displacement of K‐Ras from PDE6D. Unlike with full‐length K‐Ras‐SI (Figure S1B, Supporting Information), no clear potency differences were apparent between the four SNAP‐tagged constructs (Figure S2A, Supporting Information). Swapping of the donor and acceptor pair did not alter the results, with BRET values remaining low, suggesting a low fraction of the proteins was interacting (Figure S2B, Supporting Information). We reasoned that the low BRET signal and the difficulty resolving potency differences likely reflect the small cytosolic pool of K‐Ras available to bind PDE6D, which limits the dynamic range of the biosensor.

**Figure 2:**
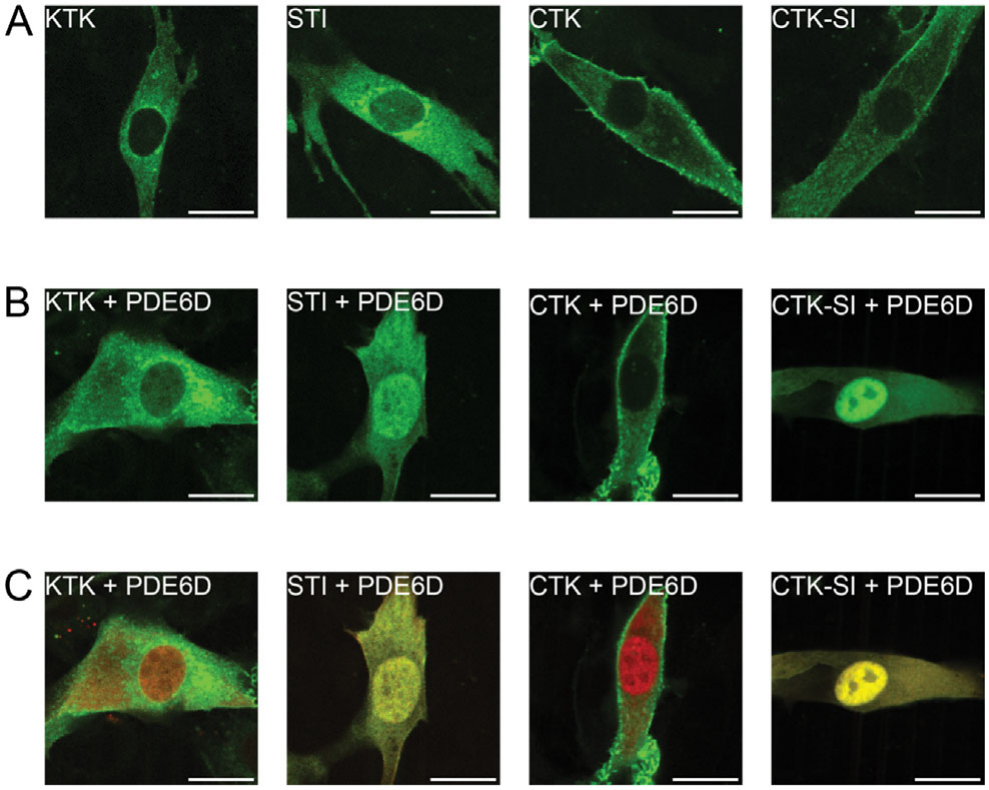
Genetically encoded PDE6D binders show distinct subcellular distributions. A–C) Confocal images of C2C12 myoblasts transfected with the (A) EGFP‐tagged variants of PDE6D binding sequences, or cotransfected with a 1:1 plasmid ratio of (B,C) EGFP‐tagged PDE6D binders and mCherry‐PDE6D. The merge of EGFP‐ and mCherry‐channels is shown in (C). The localization of mCherry‐PDE6D expressed alone is displayed in Figure S2C, Supporting Information. Scale bar = 20 µm.

Indeed, the subcellular distribution of analogous EGFP‐variants of the four unique binders varied considerably in C2C12 myoblasts, with EGFP‐CTK showing the maximum plasma membrane localization and EGFP‐STI the lowest (**Figure** **2**A). In line with the SI‐mutation increasing affinity to PDE6D and thus solubilization, coexpression of PDE6D sequestered EGFP‐STI and EGFP‐CTK‐SI to the nucleo‐cytoplasm (Figure 2B). Nuclear localization is consistent with the small size of the PDE6D/ EGFP‐binder construct complexes. The colocalization of EGFP‐STI and EGFP‐CTK‐SI with mCherry‐PDE6D becomes apparent when green‐ and red‐imaging channels are merged (Figure 2C, Figure S2C, Supporting Information).

**Figure 3:**
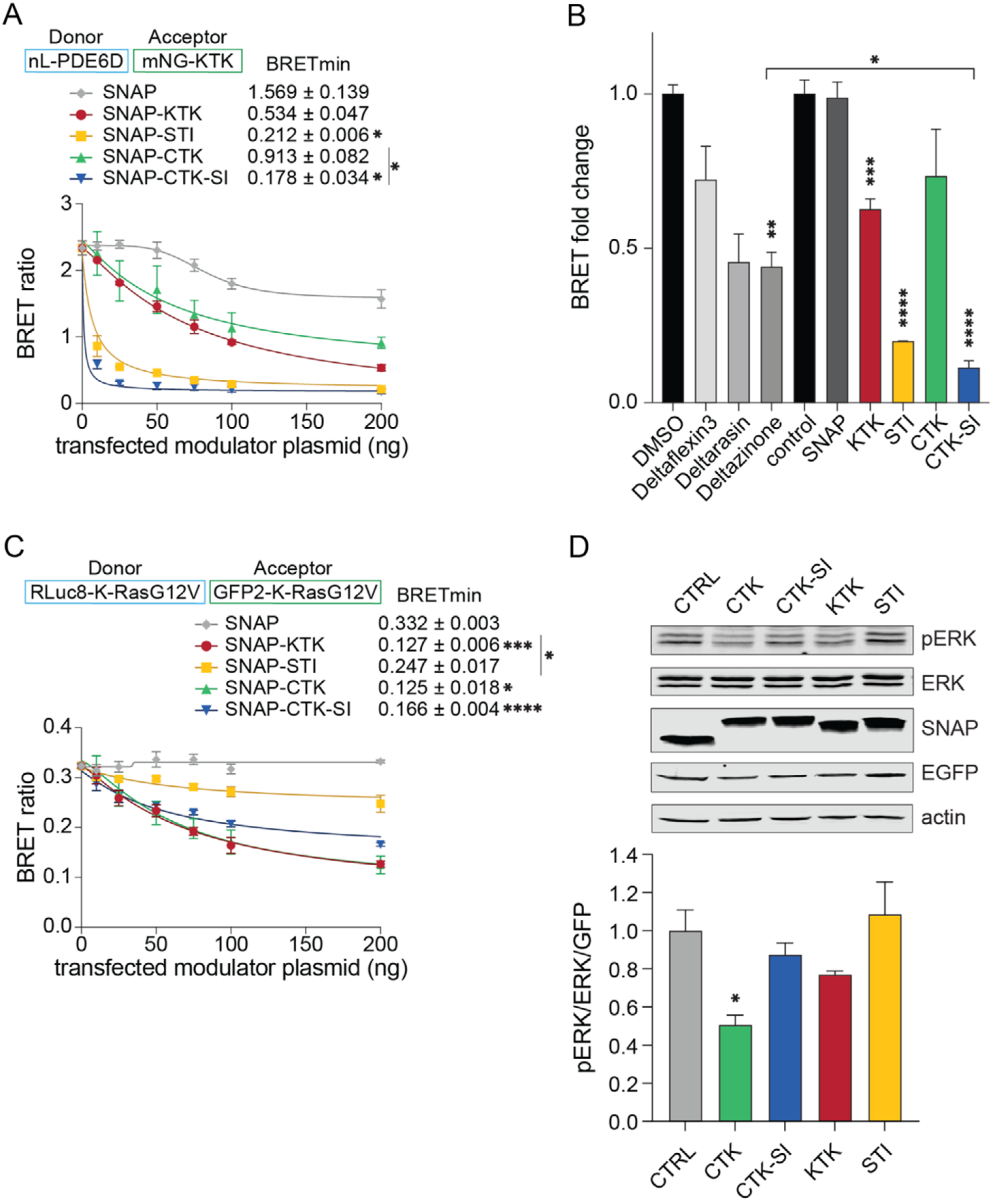
SNAP‐STI may inhibit PDE6D more effectively than small molecule inhibitors. A) Dose‐dependent inhibitory effect of SNAP‐tagged PDE6D binders on nL‐PDE6D/ mNG‐KTK BRET (donor–acceptor plasmid ratio was 1:10); means ± SEM of *n* ≥ 3 repeats are plotted. BRETmin values are the BRET values at the highest concentration of modulator and were compared between all samples with a Brown–Forsythe and Welch ANOVA analysis. B) Normalized BRET ratio of nL‐PDE6D/mNG‐KTK BRET experiments testing at 20 µM Deltaflexin3, Deltarasin, or Deltazinone and SNAP‐tagged PDE6D binders (50 ng of transfected plasmid, normalized to 0 ng of transfected modulator control); *n* ≥ 3. Means ±  SEM were plotted and compared to their respective controls or as indicated using a Brown–Forsythe and Welch ANOVA analysis. C) Dose‐dependent inhibitory effect of SNAP‐tagged constructs on RLuc8‐K‐RasG12V/ GFP2‐K‐RasG12V BRET (donor–acceptor plasmid ratio was 1:10); means ± SEM of *n* ≥ 3. BRETmin values are as in (A). D) Representative immunoblots of the phosphorylation of ERK1/2 in HEK cells transfected with mEGFP‐K‐Ras‐G12C and genetically encoded SNAP‐tagged PDE6D binders. Antibodies used for labeling are indicated. The plot shows the quantification of relative ERK phosphorylation; *n* = 4. Means ± SEM were plotted and compared to the control condition using the Kruskal–Wallis test. A *p*‐value < 0.05 was considered statistically significant, with significance levels annotated as *p ≤ 0.05; **p ≤ 0.01; ***p ≤ 0.001; ****p ≤ 0.0001.

To improve the signal and dynamic range of our PDE6D biosensor, we therefore employed a KTK‐derivative as a BRET‐acceptor, which displayed a higher cytosolic fraction able to engage with PDE6D (**Figure** **3**A). This modified BRET‐biosensor allowed a clear discrimination of PDE6D inhibitory activity, which decreased in the order STI, CTK‐SI > >KTK > CTK (Figure 3A). In line with this, direct PDE6D binding in BRET experiments with the acceptor‐tagged PDE6D binders followed the same order STI, CTK‐SI > >KTK > CTK (Figure S3A, Supporting Information).

To compare the inhibitory activity of our genetic constructs with small molecule PDE6D inhibitors, we prepared a calibration curve to estimate the approximate cytosolic concentration of SNAP‐STI and SNAP‐KTK per HEK cell (Figure S3B, Supporting Information). We thus approximated IC50(SNAP‐KTK) = 13 μM, while for SNAP‐STI, no conclusive fit could be generated, given the strong impact on the BRET already at the lowest tested concentrations (Figure 3A).

Proteins that are structurally and functionally related to PDE6D, such as UNC119A, are the most likely off‐targets of PDE6D inhibitors. We previously showed that small molecule PDE6D inhibitors can indeed target UNC119A,^[^ ^15^ ^]^ a trafficking chaperone of myristoylated proteins.^[^ ^6^ ^]^ By using our previously established UNC119A/Src‐BRET biosensor, we found that our SNAP‐tagged inhibitors do have a small activity against UNC119A (Figure S3C, Supporting Information). Considering that our inhibitors were derived from natural cargo, this may also suggest a residual promiscuity of PDE6D cargo to employ UNC119A for trafficking.

The calibration curve further allowed us to estimate that 50 ng of transfected DNA of SNAP‐tagged constructs corresponded to ≈12 μM intracellular concentration (Figure 3B, Figure S3B, Supporting Information). We were thus able to somewhat compare their activity with that of three small molecule PDE6D inhibitors, Deltaflexin3, Deltarasin, and Deltazinone, incubated at 20 μM in the cell medium.^[^ ^4^ ^,^ ^15^ ^,^ ^21^ ^]^ While the most potent small molecule inhibitor Deltazinone decreases the PDE6D/ KTK‐BRET by ≈55%, SNAP‐STI decreased it by ≈80%. When assuming that inside and outside concentrations of small molecule inhibitors are approximately the same, one can tentatively conclude that SNAP‐STI has a higher potency than tested small molecule inhibitors.

Given that the subcellular distribution of some inhibitors was similar to that of K‐Ras with partial localization to the plasma membrane (Figure 2A), we assessed disruption of K‐Ras nanoclustering. Nanoclusters are di‐/oligomeric proteo‐lipid assemblies of Ras, which are necessary for its signaling and represent a potential drug targeting opportunity.^[^ ^22^ ^]^

Using an established K‐Ras‐nanoclustering BRET assay in the absence of PDE6D overexpression,^[^ ^23^ ^,^ ^24^ ^]^ we found that inhibitors disrupted K‐Ras nanoclustering in line with their plasma membrane abundance in the order CTK, KTK > CTK‐SI > STI (Figure 3C). A decrease in the K‐RasG12V/ K‐RasG12V nanoclustering‐dependent BRET signal may also indicate an overall drop in functional K‐Ras membrane anchorage, as we demonstrated previously.^[^ ^15^ ^,^ ^25^ ^]^ At low acceptor‐to‐donor plasmid ratios, the same order was found for the proximity BRET of mNG‐derivatives with K‐Ras (Figure S3D, Supporting Information). However, at a higher ratio, CTK‐SI behaved more similarly to CTK, probably because endogenous PDE6D became saturated with CTK‐SI and could no longer sequester it, leaving it to distribute similarly to parental CTK.

Finally, inhibition of MAPK‐signaling in HEK cells (CTK > KTK, CTK‐SI > STI) (Figure 3D) followed the order of how inhibitors effectively impacted K‐Ras membrane anchorage (Figure 3C), rather than PDE6D inhibition (Figure 3A).

These data therefore indirectly support that disruption of K‐Ras nanoclustering is more potent than inhibition of PDE6D for MAPK‐activity suppression. Similarly, when comparing the impact of the inhibition of K‐Ras prenylation and thus plasma membrane anchorage using the mevalonate pathway inhibitor mevastatin, or a combination of farnesyl transferase and geranylgeranyl transferase inhibitors with that of PDE6D‐ablation, we observed a significant >60% drop in pERK‐levels with the former treatment (Figure S3E, Supporting Information), but only a ≈20% drop with nearly complete PDE6D knock‐down (Figure S3F, Supporting Information). Altogether, these data underscore that PDE6D is not a suitable surrogate target of K‐Ras‐MAPK signaling.

Our results suggest that it is possible to raise high‐affinity PDE6D inhibitors by exploiting contacts at the entrance of the prenyl‐binding pocket. The best inhibitor, SNAP‐STI, still contains a CAAX box, which instructs its farnesylation. Insertion of the farnesyl‐moiety into the lipid binding pocket provides the highest affinity contribution of PDE6D cargo and thus likely also of this inhibitor.^[^ ^17^ ^]^ The lipid binding pocket is also the target of current small molecule PDE6D inhibitors, such as Deltarasin, Deltazinone, and Deltaflexin3, which were also tested here. However, increasing the affinity of these inhibitors decreased their water solubility, as can be expected from a hydrophobic target site.^[^ ^15^ ^,^ ^26^ ^]^ In SNAP‐STI, additional contacts at the entrance of the lipid binding pocket are likely engaged, as inspired by natural cargo that modulates its activity at this site. This second site at the pocket entrance, in addition to the first site, the lipid binding pocket of PDE6D, likely allows for the superior engagement.

We developed SNAP‐STI, a bio‐relevant inhibitor that can serve as a benchmark for the cellular and in vivo assessment of small molecule PDE6D inhibitors. This will be important to resolve, for instance, if broad inhibition of PDE6D is toxic in the adult, as loss of PDE6D during development leads to the severe developmental disease Joubert‐Syndrome.^[^ ^27^ ^]^ Given that the final inhibitor is essentially a farnesylated tetrapeptide, it is plausible that a peptidomimetic with a similar potency can be developed. Indeed, others have synthesized a laurylated 11‐mer peptide as an inhibitor of UNC119, which is highly related to PDE6D.^[^ ^28^ ^]^

A plausible next step in the development of our genetically encoded inhibitors could be the testing of the synthesized STI‐CAAX‐peptide functionalized with cell‐penetrating peptides.^[^ ^29^ ^]^ Derivatives could be generated by employing non‐natural amino acids and other chemo‐synthetic building blocks that would increase the affinity at the pocket entrance, while becoming less and less reliant on the affinity contribution from within the PDE6D pocket. This would allow to remove the farnesylation instructed by the CAAX‐peptide, further reducing the overall molecular weight and potentially enabling to also drop the cell penetrating peptide, arriving at a true small molecule‐like peptidomimetic that blocks the entrance of the PDE6D binding pocket.

Already with our genetically encoded inhibitors, novel applications of PDE6D inhibition could be explored, which may include modulation of ciliary cargo proteins and the activity of the primary cilium to regulate stem cell function.^[^ ^30^ ^]^

Our results suggest that a natural cargo‐inspired affinity increase of inhibitors of PDE6D or its related trafficking chaperones has untapped potential.

## Conflict of Interest

The authors declare no conflict of interest.

## Author Contributions

**Daniel Kwaku Abankwa**: conceived the study. **Atanasio Gómez‐Mulas**: performed cloning, cell culture, BRET, and imaging experiments. **Elisabeth Schaffner‐Reckinger** and **Arnela Dautbasic**: performed cell culture and immunoblot assays. **Hanne Peeters**: performed protein purification and fluorescence polarization assays. **Rohan Chippalkatti**: supported imaging experiments. **Atanasio Gómez‐Mulas**, **Elisabeth Schaffner‐Reckinger**, **Hanne Peeters**, **Rohan Chippalkatti**, **Shehab Ismail**, and **Daniel Kwaku Abankwa**: analyzed data and wrote the paper. **Daniel Kwaku Abankwa**, **Matthew James Smith** and **Shehab Ismail**: acquired funds and supervised the project.

## Supporting information

Supplementary Material

## Data Availability

The data that support the findings of this study are available from the corresponding author upon reasonable request.
