# Supplementary material for: Development of a Genetically Encoded and Potent PDE6D Inhibitor
Source: Chembiochem. 2025 Nov 18;26(24):e202500739. doi: 10.1002/cbic.202500739 (PMC12703448; doi:10.1002/cbic.202500739)
Supplement: Supplementary file 1 — Supplementary Material [file CBIC-26-e202500739-s001.pdf]

Supporting Information for

Development of a Genetically Encoded and Potent PDE6D Inhibitor

Atanasio Gómez-Mulas, [a] Elisabeth Schaffner-Reckinger, [a] Hanne Peeters, [b] Rohan Chippalkatti, [a] Arnela Dautbasic, [a] Matthew James Smith, [c] Shehab Ismail, [b] Daniel Kwaku Abankwa, *[a]

[a] A. Gómez-Mulas, Dr. E. Schaffner-Reckinger, Dr. R. Chippalkatti, A. Dautbasic, Prof. Dr. D. K. Abankwa.
Cancer Cell Biology and Drug Discovery Group, Department of Life Sciences and Medicine
University of Luxembourg
2, place de l'Université, 4365 Esch-sur-Alzette, Luxembourg
E-mail: daniel.abankwa@uni.lu

[b] H. Peeters, Prof. Dr. S. Ismail
The Mechanistic Molecular Biochemistry Group, Department of Chemistry
KU Leuven
Celestijnenlaan 200G, Heverlee 3001, Belgium

[c] Prof. Dr. M. J. Smith.
Institute for Research in Immunology and Cancer,
Université de Montréal,
2950, Chemin de Polytechnique, Pavillon Marcelle-Coutu, Montréal, Québec, H3T 1J4, Canada
Programmes de biologie moléculaire, Université de Montréal, Montreal, Québec, H3C 3J7, Canada
Department of Pathology and Cell Biology, Faculty of Medicine, Université de Montréal, Montréal, Québec, H3T 1J4, Canada

Table of contents

Experimental Section – pages 3-6
Supporting Information Figures – pages 7-10
Supporting Information References – page 11

Experimental Methods

Peptides. Wild-type K-Ras peptide (FAM-KKKKKKSKTKC-OMe-Far) was synthesized by Biosynth and K-Ras-SI derived peptide (fluorescein-DGKKKKKKSSTIC-OMe-Far) was synthesized by JPT Innovative Peptide Solutions.

Plasmid cloning. Expression constructs were prepared using gateway recombination cloning technology (Thermo Fisher Scientific). To this end, we used gBlock gene fragments with the cDNA of the genetically encoded inhibitors (Integrated DNA Technologies), or plasmids with cDNA constructs from the Ras-Initiative (K-Ras4BG12V, K-Ras4BG12C from kit#1000000089, and PDE6D from #R702-E30), all inserts flanked by BP cloning sites. Using BP Clonase II enzyme mix, the fragments were inserted into pDONR221 plasmids to obtain entry clones flanked by LR cloning sites. Then, using the LR Clonase II enzyme mix, we combined entry clones containing the promoter, N-terminal tag, and cDNA of interest. The resulting pDest-305 plasmids were amplified in the ccdB-sensitive *E. coli* strain DH10B. Expression constructs encoding human c-Src kinase or human UNC119A have been described before [1]. All constructs were validated by Sanger sequencing.

Cell lines. C2C12 (RRID:CVCL_0188) and HEK293-EBNA (RRID:CVCL_6974) (both from ATCC) were cultured in Dulbecco's modified Eagle's medium (DMEM) containing 2 mM L-glutamine, 1 % penicillin/ streptomycin, and ~9 % (v/v) fetal bovine serum (high serum). Cells were incubated in a humidified atmosphere with 5 % CO_2 at 37 °C.

Protein purification. 6xHis-PDE6D was purified using a protocol adapted from a previously published protocol [2]. A His6-tagged PDE6D construct in a pRSF-Duet vector was transformed into BL21 DE3 *E. coli* cells. Following the transformation, precultures were grown at 37°C overnight and transferred to large cultures at 37°C until OD600 nm reached 0.5. The bacteria were then induced with 0.2 mM isopropyl β-D-1-thiogalactopyranoside (IPTG) and protein was expressed for 16 h. The bacteria were harvested by centrifugation and stored at -80°C until use. Bacterial pellets were thawed and resuspended in lysis buffer containing 50 mM Tris pH7.5, 300 mM NaCl and 2 mM Dithiothreitol (DTT) with chicken hen lysozyme before lysis using a microfluidizer at 20,000 psi. The bacterial lysate was then centrifuged at 18,600 rpm and supernatants was loaded onto a 5 mL HisTrap column (Cytiva) at 5 mL/ min. The column was then washed with 24 mM imidazole and protein was eluted using a gradient of 24-300 mM imidazole in a 50 mM Tris pH7.5, 150 mM NaCl and 0.5 mM TCEP buffer. The eluate was then passed through a Superdex HiLoad 16/600 75 pg column equilibrated with 50 mM Tris

pH7.5, 150 mM NaCl and 0.5 mM TCEP buffer at 0.7 mL/ min. 6xHis-PDE6D was then concentrated and snap-frozen in liquid nitrogen before storage at -80°C.

Fluorescence polarization peptide binding assay. Fluorescence polarization measurements were recorded on a Tecan Spark plate-reader using 96-well Corning flat black half-area non-binding plates at 22°C and using an excitation wavelength of 496 nm and emission wavelength of 524 nm for the K-Ras-derived peptide and a 498 nm excitation wavelength and 527 nm emission wavelength for the K-Ras-SI-derived peptide. All measurements were recorded in a buffer containing 50 mM Tris pH7.5, 150 mM NaCl and 0.5 mM TCEP. Fluorescence polarization was measured following a 30 min incubation of peptides at 50 nM with increasing concentrations of 6xHis-PDE6D. For the binding curve of the K-Ras-derived peptide to PDE6D, the dissociation constant was obtained by fitting the data to a quadratic equation using GraphPad Prism: FP = Fmin – (Fmin – Fmax) * (E + L + Kd-sqrt[(E + L + Kd))^2 – 4*E*L)]/(2*E). FP is the fluorescence polarization signal, Fmin and Fmax is the minimum and maximum polarization signal, E is the K-Ras peptide concentration, L is the 6xHis-PDE6D concentration and K_d is the dissociation constant as a measure for affinity. For the active site titration of K-Ras-SI-derived peptide to PDE6D, the linear regimes were fitted with a linear equation Y=slope*L+intercept.

Bioluminescence Resonance Energy Transfer (BRET). BRET experiments were conducted as previously described [3]. Briefly, 220,000 HEK293-EBNA cells were seeded per well in 12-well plates, transfected after 24 h with the plasmid constructs using 2 μL of JetPrime, and measured 48 h later. Donor saturation titration BRET was performed as previously described by us. From these data, we determined the pseudolinear regime for the optimal acceptor-to-donor ratio in dose-response experiments. These latter experiments were conducted by adding increasing amounts of a modulator (plasmid or compound) 24 h after transfection, measuring 16 h later. BRET was measured using a CLARIOstar Microplate Reader (BMG Labtech) using white flat-bottom 96-well plates as described [3]. Donor saturation data were fitted to a one-phase association curve, while dose-response data were fitted to a [inhibitor] vs. response - variable slope (four parameters). IC50 values from BRET experiments were derived from the fitted curves, as described before [3b].

Confocal microscopy. C2C12 cells were seeded in 6-well plates with 0.17 mm coverslips at 200,000 cells per well. After 24 h, the cells were transfected with JetPrime, and 4 h later, fresh high serum medium was added to the cells. 48 h after transfection, the cells were fixed with 4

% (w/v) paraformaldehyde in PBS and permeabilized with 0.5 % Triton X-100. The nuclei were stained with 0.2 µg/ mL Hoechst 33342 in PBS containing 0.05 % v/v Tween 20 and then washed. Vectashield mounting medium was added to the glass slides, and the coverslips were mounted on them. The fixed cells on coverslips were imaged using a 60 × NA 1.3 oil immersion objective on a Nikon Ti-E microscope equipped with a Yokogawa CSU-W1 spinning disk confocal unit and an Andor iXon Ultra EMCCD camera. The EGFP-fluorophore was detected using a 488 nm laser for excitation and a 535/20 nm band pass filter. The mCherry fluorescence was detected using a 561 nm laser and a 560/40 band pass filter. Z-stacks were acquired with 0.6 µm spacing. Images were acquired with Nikon NIS-elements software and analyzed in Fiji/ ImageJ.

Immunoblotting. Prior to transient transfection, 100,000 HEK293-EBNA cells were seeded in 2 mL DMEM/ well in 6-well plates. After 24 h, cells were transfected with 100 nM siRNA as reported before [1], and 3.75 µL Lipofectamine RNAiMAX, and after 48 h, transfection was performed with 0.5 µg of each plasmid DNA and 3 µL Lipofectamine 2000. In situ cell lysis was performed 24 h after plasmid transfection as described before in ice-cold lysis buffer (50 mM Tris-HCl pH 7.5, 150 mM NaCl, 0.1 % w/v SDS, 5 mM EDTA, 1 % v/v Nonidet P-40, 1 % v/v Triton X-100, 1 % w/v sodium-deoxycholate, 1 mM Na_3VO_4, 10 mM NaF, 100 µM leupeptin) containing cocktails of protease inhibitors and phosphatase inhibitors [1]. After clarification of the lysates by centrifugation, the total protein concentration was determined by performing a Bradford assay. A standard curve with bovine serum albumin was established. SDS-PAGE was performed using 10 % w/v homemade polyacrylamide gels to resolve proteins (40 µg per lane) under reducing conditions. Proteins were then transferred by semi-dry transfer onto nitrocellulose membranes. Saturation was performed for 1 h at 22 °C in PBS containing 2 % w/v BSA and 0.2 v/v % TWEEN 20, and membranes were incubated overnight at 4 °C with primary antibodies diluted in saturation buffer. Incubation with corresponding secondary antibodies diluted in saturation buffer was carried out for 1 h at 22 °C. At least three wash steps in PBS 0.2 % TWEEN 20 were performed after each antibody incubation. For each blot, *β*-actin levels were determined as a loading control. An Odyssey Infrared Image System (LI-COR Biosciences) was used to quantify signal intensities. ERK phosphorylation was calculated as described before [1], and data were scaled to the corresponding control present on each blot.

Estimation of the expression of SNAP-tagged peptides in cells. On immunoblots, band intensities corresponding to SNAP-tagged peptides encoded by the PDE6D binder constructs

were determined using an anti-SNAP antibody and were expressed as ratios vs. a known amount of purified SNAP protein (New England Biolabs) loaded on the same gel. Considering the approximate number of cells loaded per well and using an approximate HEK293-EBNA cell volume of 2.5 pL [4], a dose response curve of the concentration of SNAP-tagged peptide per cell vs. the amount of transfected DNA was established. Using Graph Pad Prism software, simple linear regression was performed to obtain the linear fit of the dose-response curve, and the R^2 value was determined. Thus, we derived the following formula to calculate the approximate intracellular concentration (c_{ic}), of SNAP-STI and SNAP-CTK based on their transfected plasmid amounts ($m_{plasmid}$): c_{ic} [μM] = 0.08406 [μM/ng] * $m_{plasmid}$ [ng] + 8.689 [μM].

Statistical analysis. Graph Pad Prism software was used to analyze data. Unless otherwise indicated, data plots show mean values ± SEM. The number of independent biological repeats is indicated by n. A Brown-Forsythe and Welch ANOVA analysis was performed comparing the BRET ratio value of the highest modulator amount (BRETmin) between all samples, or the highest A/D plasmid ratio values (BRETtop) between all samples, unless stated otherwise in the legends. A p-value < 0.05 was considered statistically significant, with significance levels annotated as: *$p \leq 0.05$; **$p \leq 0.01$; ***$p \leq 0.001$; ****$p \leq 0.0001$.

Supporting Information Figures

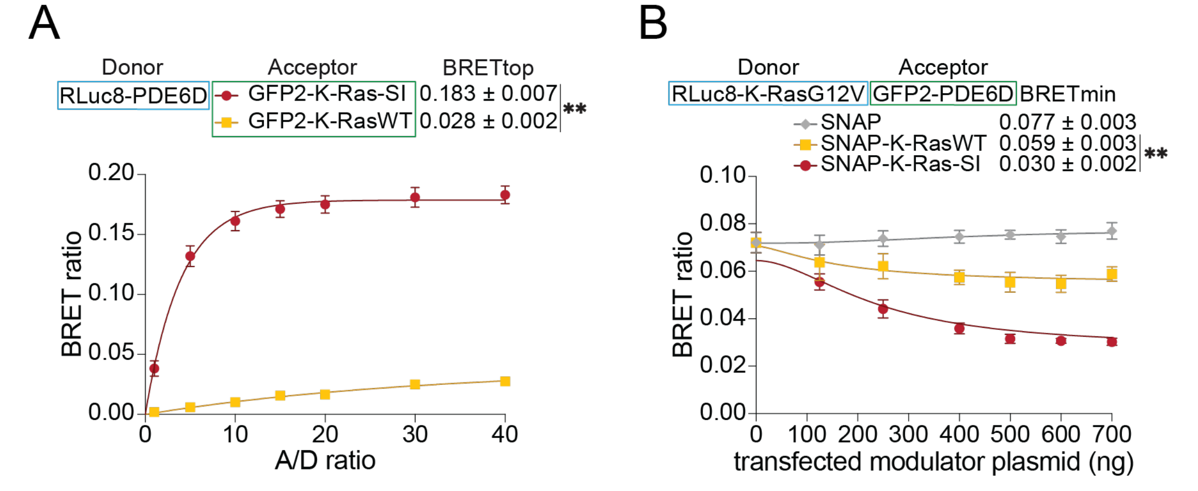

Figure S1. Data supplementing main Figure 1. A) BRET donor saturation titration curves of 25 ng RLuc8-PDE6D donor plasmid titrated with increasing amounts of acceptor plasmid GFP2-K-RasWT, or GFP2-K-Ras-SI. A/D ratio gives the acceptor-to-donor plasmid ratio; mean ± SEM of n ≥ 3 repeats are plotted. BRETtop values, which are the BRET-values at highest A/D ratio, were compared with a two-tailed Welch's t-test. **B**) Dose-dependent effect of the SNAP-tagged PDE6D binders on RLuc8-K-RasG12V/ GFP2-PDE6D BRET (donor-acceptor plasmid ratio = 1:10); means ± SEM of n ≥ 3 repeats are plotted. BRETmin values were compared between all samples with a Brown-Forsythe and Welch ANOVA analysis.

A p-value < 0.05 was considered statistically significant, with significance levels annotated as: $^{**}p \leq 0.01$.

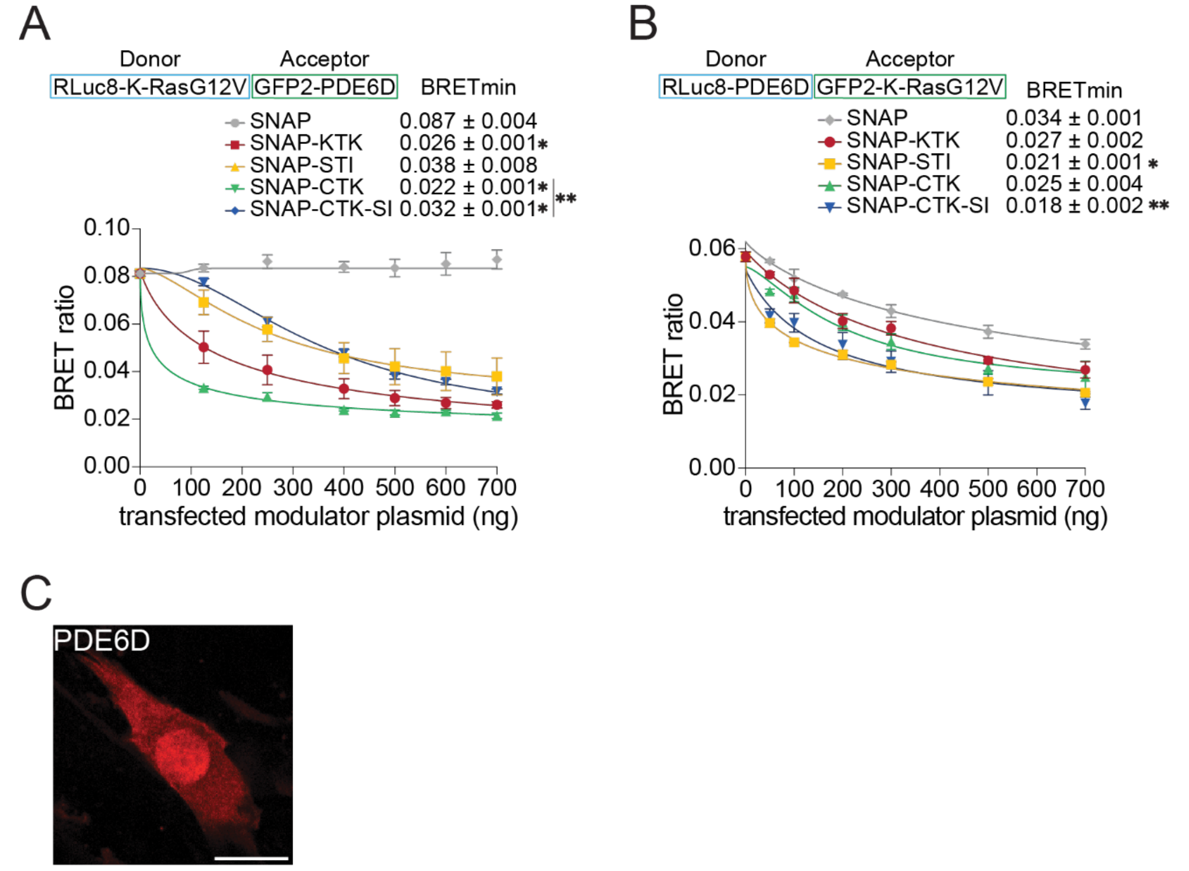

Figure S2. Data supplementing main Figure 2. A, B) Dose-dependent effect of the SNAP-tagged PDE6D binders on RLuc8-K-RasG12V/ GFP2-PDE6D BRET (donor-acceptor plasmid ratio = 1:10; n ≥ 3) (A), or on RLuc8-PDE6D/ GFP2-K-RasG12V BRET (donor-acceptor plasmid ratio = 1:20; n ≥ 3) (B). Means ± SEM are plotted. BRETmin values were compared between all samples with a Brown-Forsythe and Welch ANOVA analysis. **C)** Confocal image of C2C12 myoblasts transfected with mCherry-tagged PDE6D alone. Scale bar = 20 μm.

A p-value < 0.05 was considered statistically significant, with significance levels annotated as: *$p \leq 0.05$; **$p \leq 0.01$.

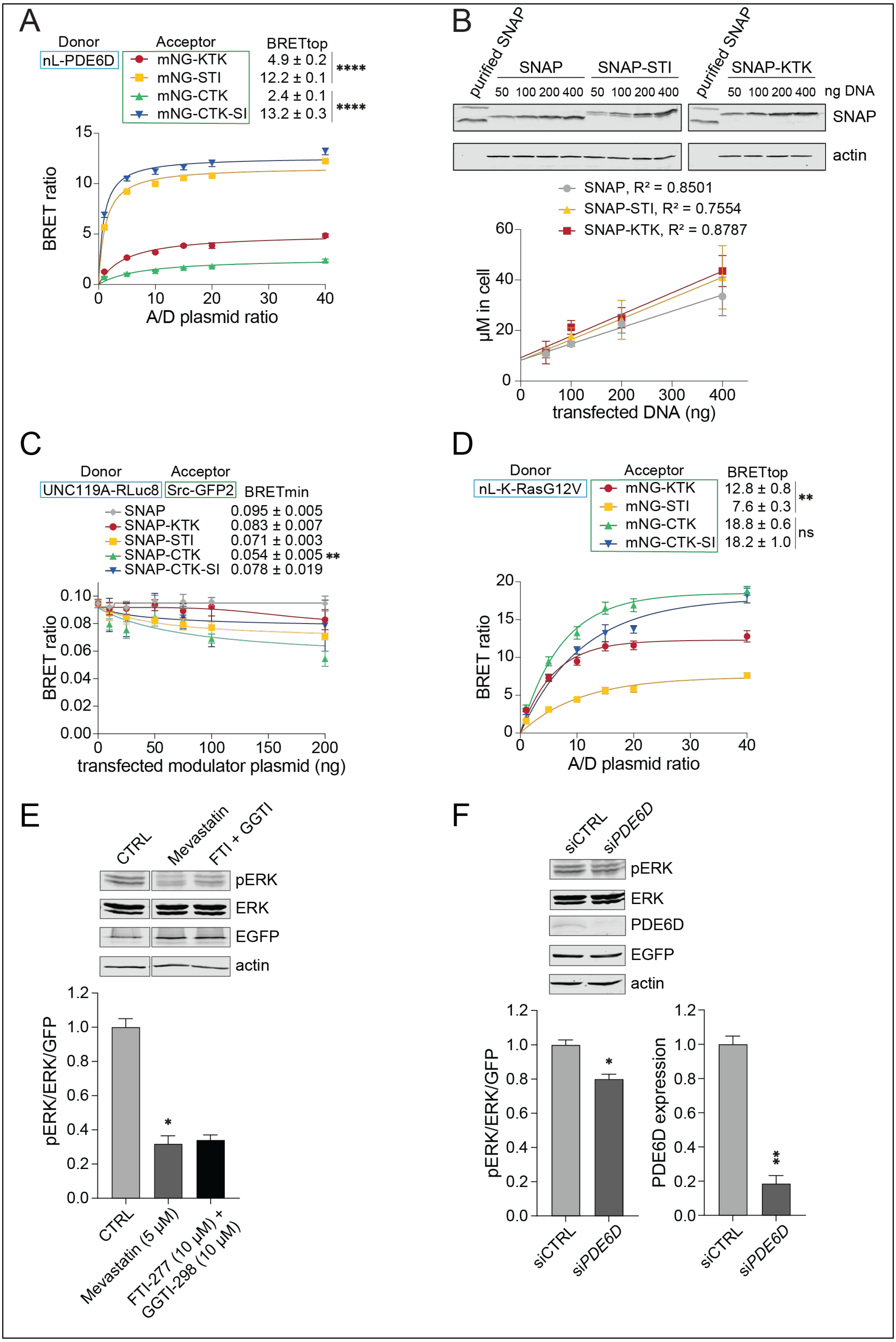
A
Donor
nL-PDE6D
Acceptor
mNG-KTK
mNG-STI
mNG-CTK
mNG-CTK-SI
BRETtop
4.9 ± 0.2
12.2 ± 0.1
2.4 ± 0.1
13.2 ± 0.3
****
****
BRET ratio
A/D plasmid ratio
B
purified SNAP
SNAP
SNAP-STI
SNAP-KTK
50 100 200 400
ng DNA
actin
SNAP, R² = 0.8501
SNAP-STI, R² = 0.7554
SNAP-KTK, R² = 0.8787
µM in cell
transfected DNA (ng)
C
Donor
UNC119A-RLuc8
Acceptor
Src-GFP2
BRETmin
SNAP 0.095 ± 0.005
SNAP-KTK 0.083 ± 0.007
SNAP-STI 0.071 ± 0.003
SNAP-CTK 0.054 ± 0.005 **
SNAP-CTK-SI 0.078 ± 0.019
BRET ratio
transfected modulator plasmid (ng)
D
Donor
nL-K-RasG12V
Acceptor
mNG-KTK
mNG-STI
mNG-CTK
mNG-CTK-SI
BRETtop
12.8 ± 0.8
7.6 ± 0.3
18.8 ± 0.6
18.2 ± 1.0
**
ns
BRET ratio
A/D plasmid ratio
E
CTRL
Mevastatin
FTI + GGTI
pERK
ERK
EGFP
actin
pERK/ERK/GFP
CTRL
Mevastatin (5 µM)
FTI-277 (10 µM) + GGTI-298 (10 µM)
F
siCTRL
siPDE6D
pERK
ERK
PDE6D
EGFP
actin
pERK/ERK/GFP
PDE6D expression
siCTRL
siPDE6D

Figure S3. Data supplementing main Figure 3. A) BRET donor saturation titration curves of 25 ng nL-PDE6D with increasing amounts of the mNG-tagged PDE6D binder constructs; mean ± SEM of n = 5 repeats represented. BRETtop values were compared between all samples with a Brown-Forsythe and Welch ANOVA analysis. **B**) Representative immunoblots showing the expression of SNAP-KTK and -STI, and 2.5 *μ*g purified SNAP protein loaded on the same gel. SNAP corresponds to the lower band in the lane of purified SNAP. Antibodies used for labeling are indicated. The calibration curves show the estimated concentration of SNAP-tagged constructs per cell vs. the amount of transfected DNA. Mean ± SD are shown. A simple linear regression was fitted to the data, and the R^2 value was determined. **C**) Dose-dependent effect of the SNAP-tagged PDE6D binders on UNC119A-RLuc8/ Src-GFP2 BRET (donor-acceptor plasmid ratio = 1:10); n ≥ 4. BRETmin values were compared between all samples with a Brown-Forsythe and Welch ANOVA analysis. **D**) BRET donor saturation titration curves of 25 ng nL-K-RasG12V donor plasmid with increasing amounts of the mNG-tagged PDE6D binder constructs; n = 5. **E, F**) Immunoblot analysis of the phosphorylation of ERK1/2 (pERK) in HEK cells transfected with EGFP-K-Ras-G12C and treated overnight with Mevastatin or a combination of farnesyl transferase inhibitor FTI-277 and geranylgeranyl transferase inhibitor GGTI-298 (E) or following siRNA-mediated PDE6D downregulation (F). Antibodies used for labeling are indicated in representative immunoblots. The plots show the quantification of relative ERK phosphorylation or PDE6D expression; n = 4. Statistical analysis as compared to the control condition was performed with the Kruskal-Wallis test (E) or the Mann-Whitney test (F).

A p-value < 0.05 was considered statistically significant, with significance levels annotated as: **p* ≤ 0.05; ***p* ≤ 0.01; *****p* ≤ 0.0001.

Supporting Information References

[1] P. Kaya, E. Schaffner-Reckinger, G. B. Manoharan, V. Vukic, A. Kiriazis, M. Ledda, M. Burgos Renedo, K. Pavic, A. Gaigneaux, E. Glaab, D. K. Abankwa, *J Med Chem* **2024**, *67*, 8569-8584.

[2] T. Yelland, E. Garcia, C. Parry, D. Kowalczyk, M. Wojnowska, A. Gohlke, M. Zalar, K. Cameron, G. Goodwin, Q. Yu, P. C. Zhu, Y. ElMaghloob, A. Pugliese, L. Archibald, A. Jamieson, Y. X. Chen, D. McArthur, J. Bower, S. Ismail, *J Med Chem* **2022**, *65*, 1898-1914.

[3] aC. J. Duval, C. L. Steffen, K. Pavic, D. K. Abankwa, *STAR Protoc* **2024**, *5*, 103348; bC. L. Steffen, G. B. Manoharan, K. Pavic, A. Yeste-Vazquez, M. Knuuttila, N. Arora, Y. Zhou, H. Harma, A. Gaigneaux, T. N. Grossmann, D. K. Abankwa, *Commun Biol* **2024**, *7*, 837.

[4] D. Abankwa, H. Vogel, *J Cell Sci* **2007**, *120*, 2953-2962.
